# Attitudes towards Electronic Cigarettes Regulation in Indoor Workplaces and Selected Public and Private Places: A Population-Based Cross-Sectional Study

**DOI:** 10.1371/journal.pone.0114256

**Published:** 2014-12-03

**Authors:** Jose M. Martínez-Sánchez, Montse Ballbè, Marcela Fu, Juan C. Martín-Sánchez, Mark Gottlieb, Esteve Saltó, Constantine I. Vardavas, Richard Daynard, Gregory N. Connolly, Esteve Fernández

**Affiliations:** 1 Tobacco Control Unit, Cancer Prevention and Control Program, Institut Català d’Oncologia, L’Hospitalet de Llobregat, Barcelona, Spain; 2 Cancer Prevention and Control Group, Institut d’Investigació Biomèdica de Bellvitge - IDIBELL, L’Hospitalet de Llobregat, Barcelona, Spain; 3 Biostatistics Unit, Department of Basic Sciences, Universitat Internacional de Catalunya, Sant Cugat del Vallès, Spain; 4 Public Health Advocacy Institute, Northeastern University School of Law, Boston, Massachusetts, United States of America; 5 Department of Clinical Sciences, Universitat de Barcelona, Barcelona, Spain; 6 Addictions Unit, Institute of Neurosciences, Hospital Clínic de Barcelona - IDIBAPS, Barcelona, Spain; 7 Health Plan Directorate, Ministry of Health, Generalitat de Catalunya, Spain; 8 Department of Public Health, Universitat de Barcelona, Barcelona, Spain; 9 Department of Social and Behavioral Sciences, Center for Global Tobacco Control, Harvard School of Public Health, Boston, Massachusetts, United States of America; The University of Auckland, New Zealand

## Abstract

**Background:**

Currently, there is an intensive debate about the regulation of the use of electronic cigarettes (e-cigarettes) in indoor places. The aim of this study was to assess the attitudes toward e-cigarette use in indoor workplaces and selected public and private venues among the general population in Barcelona (Spain) in 2013–2014.

**Methods:**

This is a cross-sectional study of a representative sample of the population of Barcelona (n = 736). The field work was conducted between May 2013 and February 2014. We computed the prevalence and the adjusted odds ratios (OR) derived from multivariable logistic regression models.

**Results:**

The awareness of e-cigarettes was 82.3%. Forty five percent of respondents did not agree with the use of e-cigarettes in public places and 52.3% in workplaces. The proportion of disapproval of the use of e-cigarettes in indoor places was higher at 71.5% for schools and 65.8% for hospitals and health care centers; while the prevalence of disapproval of e-cigarette use in homes and cars was lower (18.0% and 32.5%, respectively). Respondents who disagreed on the use of e-cigarettes in indoor workplaces were more likely to be older (OR = 1.64 and 1.97 for groups 45–64 and ≧65 years old, respectively), those with a high educational level (OR = 1.60), and never and former smokers (OR = 2.34 and 2.16, respectively). Increased scores in the Fagerström test for cigarette dependence were also related to increased support for their use.

**Conclusions:**

Based on this population based study, half of the general population of Barcelona does not support the use of e-cigarettes in indoor workplaces and public places, with the percentage reaching 65% for use in schools, hospitals and health care centers. Consequently, there is good societal support in Spain for the politicians and legislators to promote policies restricting e-cigarettes use in workplaces and public places, including hospitality venues.

## Introduction

Secondhand smoke (SHS) exposure is responsible of 1% (603,000 deaths per year) of mortality worldwide [1]. Several countries have implemented smoke-free bans in all indoor workplaces and public places in order to protect the non-smoker population, including children, from the harmful health effects of the SHS exposure as noted by Article 8 of the World Health organization, Framework Convention on Tobacco Control [2]. Scientific evidence has shown that these bans reduce the SHS exposure and the burden of disease among non-smokers [3]. Particularly, smoke-free legislation has been associated with a decrease in hospital admissions for cardiovascular and respiratory diseases among adults [4], and with a reduction of preterm birth and hospitality attendance for asthma among children [5].

Since 2007, the popularity of electronic cigarettes (e-cigarettes) has grown rapidly around the world. Among the general population of the United States (US) [6] the prevalence of ever-use, according to a web-based survey, showed a twofold increase between 2010 and 2011 (from 3.3% to 6.2%). This double increase was also observed in US adolescents [7] and in middle and high school students [8] between 2011 and 2012. In Europe, there is certain variability in the prevalence of use among studies, depending on the population and the questions used in the surveys [9]–[12]. According to a secondary analysis of the 2012 Eurobarometer, 7% of the European citizens tried the e-cigarettes with experimentation significantly higher among current smokers [13]. Moreover, the use of e-cigarettes with nicotine is estimated in 62.5% among ever-users of e-cigarettes according to a study conducted in a representative sample of the general population in Spain [12].

There are two key messages that are promoted by a number of e-cigarettes companies to promote their use: 1) their utility to quit or to reduce the tobacco consumption, and 2) the possibility to use them in workplaces and other public places where smoking is not allowed. According to a systematic review of e-cigarettes’ advertisements in websites, the main message highlights their health benefits, whereas 88% claimed that e-cigarettes could be used anywhere [14].

The rise of e-cigarette use and the ability to use them anywhere, due to a regulatory void, is hypothesized to potentially be a gateway to renormalize smoking in indoor public places. Moreover, as first generation disposable e-cigarettes look like conventional cigarettes, thus they could imply the message that smoking is an accepted behavior. Hence, health researchers, legislators, and some regulatory agencies have suggested that the use of e-cigarettes in workplaces and other public places should be prohibited, as is done with combustible tobacco products.

To our knowledge, there is lack of evidence about public support for e-cigarette use in indoor workplaces and other public venues. The objective of this study is to assess the support and correlates of e-cigarette use in indoor workplaces and selected public and private venues among the general population in Barcelona (Spain) in 2013–2014.

## Methods

The Determinants of Cotinine phase 3 project (dCOT3, website: http://bioinfo.iconcologia.net/es/content/estudio-dcot3) is a longitudinal study of a representative sample of the adult (≧16 years old) non-institutionalized population of the city of Barcelona (Spain) (n = 1245, 694 women and 551 men). The baseline survey was conducted in 2004–2005 and its detailed design is provided elsewhere [15], [16]. We followed-up all the adult participants who responded to the face-to-face questionnaire in 2004–2005 and agreed to participate in a new study in the future. The ethics committee of the Bellvitge University Hospital approved the study protocol and the written informed consent. All participants signed the written informed consent. At the beginning of 2013, we did a linkage with the Insured Central Registry of Catalonia (Registre Central d’Assegurats, RCA) in order to update the vital status and contact information (addresses and telephone numbers) of all participants. We restricted the follow-up to the participants who continued living in the city of Barcelona and its province in 2013.

For the present study we restricted the analysis to this cross-sectional data. We traced 1,010 people out of the 1,245 participants in the baseline study using the RCA (101 have died, 49 migrated out of the province of Barcelona, and 85 did not give consent to be followed or were <18 years old in 2004–2005). In February 2013, we sent a letter informing about the main results of the study of 2004–05 and that an interviewer would visit them at home to administer another face-to-face questionnaire. The follow-up survey was conducted between May 2013 and February 2014. 72.9% agreed to participate, 18.5% refused participation, 7.2% were not localized, and 1.3% had died. The final sample analyzed was 736 individuals (336 men and 400 women). There were no statistically significant differences between the participants and the people lost in the follow-up according to sex, level of education, and smoking status. However, the final sample overestimates the older people compared to the current distribution of the population in Barcelona. For this reason, we weighted the data according to the age distribution of Barcelona in 2013.

We assumed that participants were aware of e-cigarettes when they answered affirmatively the question: “Do you know what an e-cigarette is?” We also gathered information on ever-use of e-cigarettes using the question: “Have you ever used e-cigarettes?” The possible answers to this question were: “yes, currently”, “yes, in the past”, “I have only tried e-cigarettes”, and “I have never used e-cigarettes”. We considered ever-users of e-cigarettes those people who answered “yes, currently”, “yes, in the past” and “I have only tried e-cigarettes”.

Information on support to the use of e-cigarettes in indoor venues was asked only to those who were aware of them (82.3% of the sample: 606 participants). We used the following question: “To what extent do you agree or disagree with allowing the use of e-cigarettes in the following indoor settings?” The indoor settings considered were: all public places, workplaces, hospitals and other health care centers, schools, the hospitality sector (bars, restaurants pubs, and nightclubs), public transports, taxis, planes, and private venues (home and private vehicles). The possible answers for these questions were: “totally agree”, “agree”, “neither agree nor disagree”, “disagree”, and “totally disagree”. We considered that participants supported the regulation of e-cigarette use in the different venues studied when they answered “totally disagree” or “disagree”. Finally, we asked about the support for the sale of e-liquids containing nicotine using the question: “Do you agree with the marketing of e-cigarettes with nicotine?” The answer for this question was also: “totally agree”, “agree”, “neither agree nor disagree”, “disagree”, and “totally disagree”. We considered participants who opposed to the sale and marketing of e-cigarettes with nicotine when they answered “totally disagree” or “disagree”.

We calculated the proportion of people according to the attitudes towards e-cigarette regulation. We fitted multivariable logistic regression models adjusted for sex, age, and educational level to calculate the odds ratios (OR) with their 95% confidence intervals (CI). All analyses were stratified for sex, groups of age (≤44 years old, 45–64 years old, and ≧65 years old), educational level -categorized as low (no qualification up to middle school diploma), intermediate (high school), and high (university degree)-, cigarette smoking status (current smokers, former smokers, and never smokers), ever e-cigarette users (yes and no), and level of nicotine dependence measured with the Fagerström test for cigarette dependence (FTCD) [17] for current cigarette smokers (categorized into low-medium dependence for scores between 0 and 5, and high dependence for scores between 6 and 10) [18]. We used SPSS v.21 for all the statistical analyses.

## Results

Awareness of e-cigarettes among the study population was 82.3% (95%CI: 79.5–85.1). There were statistically significant differences between people aware and not aware of e-cigarettes according to age, educational level, and current smoking status. Higher levels of awareness were found among younger people, higher education levels, and current smokers (table 1).

**Table 1:** Socio-demographic differences between the people aware and not aware of e-cigarettes.

		Yes, I have heard of electronic cigarettes	No, I have not heard of electronic cigarettes	
		n = 606	n = 130	p-value
Sex				0.141*
Men		47.5 (43.5−51.5)	40.5 (32.1−48.9)	
Women		52.5 (48.5−56.5)	59.5 (51.1−67.9)	
Age				<0.001*
≤44 years old		46.3 (42.3−50.3)	13.8 (7.9−19.8)	<0.001**
45–64 years old		35.7 (31.9−39.5)	17.7 (11.1−24.3)	
≥65 years old		18.0 (15.0−21.1)	68.5 (60.5−76.4)	
Educational level				
Low		11.7 (9.2−14.3)	45.8 (37.2−54.4)	<0.001*
Intermediate		38.9 (35.1−42.8)	31.3 (23.3−39.3)	<0.001**
High		49.3 (45.4−53.3)	22.9 (15.7−30.1)	
Smoking status				<0.001*
Never smoker		35.3 (31.5−39.1)	60.3 (51.9−68.7)	
Former smoker		34.7 (30.9−38.4)	32.1 (24.0−40.1)	
Current smoker		30.0 (26.4−33.7)	7.6 (3.1−12.2)	

*Chi square **Chi square test for trend.

Table 2 shows the proportion of people who disagreed with the use of e-cigarettes in indoor workplaces, selected public places, and in hospitality venues (those who answered to “disagree” or “totally disagree” with using e-cigarettes). Overall, 45.0% disagreed with the use of e-cigarettes in any indoor public place, with the lack of support for their use. This figures were significantly higher among older people (OR = 1.64 and 1.97 for groups 45–64 and ≧65 years old, respectively), those with a high educational level (OR = 1.60), and among never and former smokers (OR = 2.34 and 2.16, respectively). The lowest percentage of disagreement for using e-cigarette in all public places was found among current smokers with high scores in the FTCD (17.9%) and among the ever e-cigarette users (27.6%). The percentage of people who disagreed with the use of e-cigarettes in indoor workplaces was 52.3%, which was statistically significantly higher among non-smokers (never and former), smokers with low-medium cigarette dependence, and never e-cigarette users (table 2). The highest percentages of disagreement with the use of e-cigarettes were found for schools and hospitals and health care centers (71.5% and 65.8%, respectively). The percentage was 46.7% for hospitality venues (bars, restaurants, pubs, and nightclubs) (table 2).

**Table 2:** Percentages (%), odds ratio (OR), and 95% confidence interval (95%CI) of people who disagree with allowing the use of electronic cigarettes (who were “disagree” and “totally disagree” with their use) in workplaces, some public places, and hospitality venues.

		t		Workplaces		Hospitals and otherhealth care centers		Schools		Bars andrestaurants		Pubs andnightclubs	
	n	%	OR (95%CI)	%	OR (95%CI)	%	OR (95%CI)	%	OR (95%CI)	%	OR (95%CI)	%	OR (95%CI)
Overall	606	45.0	–	52.3	–	65.8	–	71.5	–	46.7	–	46.8	–
Sex													
Men	288	41.1	1	50.0	1	60.2	1	65.9	1	42.8	1	43.7	1
Women	318	48.3	1.32 (0.94−1.87)	54.4	1.19 (0.85−1.66)	70.8	1.64 (1.14−2.35)	76.6	1.77 (1.21−2.58)	50.2	1.31 (0.93−1.84)	49.5	1.23 (0.88−1.73)
Age													
≤44 years old	280	39.9	1	52.1	1	64.0	1	69.3	1	45.1	1	46.1	0.88 (0.53–1.45)
45–64 years old	216	49.2	1.64 (1.11−2.42)	53.6	0.87 (0.52−1.44)	67.5	1.35 (0.89−2.02)	74.6	1.45 (0.94−2.24)	49.0	1.28 (0.87−1.87)	48.2	1.03 (0.62–1.72)
≥65 years old	109	50.0	1.97 (1.17−3.33)	51.0	1.02 (0.61−1.70)	67.0	1.78 (1.03−3.10)	71.0	1.64 (0.92−2.91)	47.0	1.27 (0.76−2.10)	46.0	1
Educational level													
Low	71	41.2	0.82 (0.45−1.47)	47.1	0.98 (0.55−1.74)	50.0	1	55.9	1	45.6	1.18 (0.66−2.10)	45.6	1.20 (0.68−2.14)
Intermediate	236	40.6	1	46.5	1	63.1	2.18 (1.20−3.95)	71.2	2.46 (1.34−4.51)	39.8	1	40.3	1
High	299	49.4	1.60 (1.09−2.33)	58.2	1.63 (1.12−2.35)	71.8	3.47 (1.89−6.38)	75.6	3.27 (1.76−6.09)	52.4	1.72 (1.19−2.50)	52.4	1.67 (1.15−2.41)
Smoking status													
Never smoker	214	52.0	2.34 (1.50−3.64)	61.1	2.32 (1.51−3.56)	72.1	2.58 (1.65−4.04)	78.4	2.87 (1.79−4.61)	54.0	2.56 (1.65−3.98)	54.9	2.71 (1.74−4.22)
Former smoker	210	51.4	2.16 (1.38−3.40)	54.7	1.77 (1.14−2.73)	74.6	2.95 (1.85−4.70)	77.8	2.76 (1.70−4.49)	53.9	2.59 (1.65−4.06)	53.4	2.59 (1.66−4.06)
Current smoker	182	30.0	1	39.5	1	48.9	1	55.9	1	30.4	1	30.4	1
FTCD													
Low-Medium (0–5)	150	32.4	2.30 (0.77−6.80)	44.1	3.42 (1.24−9.42)	52.1	2.48 (0.99−6.22)	57.4	1.75 (0.71−4.31)	34.5	4.77 (1.34−16.92)	34.5	4.77 (1.34−16.92)
High (6–10)	32	17.9	1	20.0	1	33.3	1	46.7	1	10.3	1	10.3	1
Ever use of e-cigarettes													
No	546	47.0	1.94 (1.05−3.59)	54.6	2.24 (1.25−4.00)	67.9	1.92 (1.09−3.39)	73.1	1.85 (1.03−3.32)	48.8	1.97 (1.08−3.56)	49.2	2.43 (1.32−4.47)
Yes	60	27.6	1	33.3	1	48.3	1	56.9	1	30.0	1	26.7	1

All ORs were adjusted for sex, age, and educational level.

FTCD: Fagerström test for cigarette dependence. OR: Odd Ratio; CI: confidence intervals.

The lowest percentages of disagreement for using e-cigarettes were found for private venues (18.0% at homes and 32.5% in private vehicles). Men, young people (≤44 years old), and people with intermediate educational level showed the least disagreement with the e-cigarette use in private venues (table 3). The percentage of disagreement with the use of e-cigarette in public transport, taxis, and planes was around 60% (table 3). Overall, we found less disagreement for using e-cigarettes in all public and private venues among current smokers, particularly among smokers with high scores in FTCD, and ever e-cigarettes users (table 2 and 3).

**Table 3:** Percentages (%), odds ratio (OR), and 95% confidence interval (95%CI) of people who disagree with allowing the use of electronic cigarettes (who are “disagree” and “totally disagree” with their use) in homes, private vehicles, and public transports.

			Home		Private vehicles		Public transport		Taxis		Planes	
	n		%	OR(95%CI)	%	OR(95%CI)	%	OR(95%CI)	%	OR(95%CI)	%	OR(95%CI)
Overall	606		18.0	–	32.5	–	59.8	–	58.4	–	59.5	–
Sex												
Men	288		16.9	1	30.5	1	54.9	1	53.8	1	54.5	1
Women	318		19.2	1.12(0.72−1.73)	34.2	1.16(0.81−1.67)	64.3	1.49(1.05−2.10)	62.8	1.47(1.04−2.07)	64.1	1.511(1.07−2.13)
Age												
≤44 years old	280		13.8	1	24.7	1	57.3	1	56.3	1	58.6	1
45–64 years old	216		23.4	2.01(1.23−3.29)	38.5	2.03(1.34−3.06)	62.2	1.37(0.92−2.02)	60.2	1.28(0.87−1.89)	60.7	1.20(0.81−1.77)
≥65 years old	109		18.6	1.48(0.76−2.86)	42.9	2.48(1.46−4.23)	61.6	1.62(0.96−2.75)	61.0	1.64(0.97−2.77)	60.0	1.48(0.88−2.50)
Educational level												
Low	71		23.2	1.52(0.75−3.09)	38.2	1.17(0.64−2.14)	50.7	1	47.8	1	47.1	1
Intermediate	236		15.5	1	29.5	1	57.1	1.56(0.87−2.80)	56.2	1.70(0.95−3.04)	57.1	1.83(1.02−3.28)
High	299		18.6	1.42(0.87−2.31)	33.2	1.41(0.95−2.11)	64.3	2.25(1.24−4.07)	62.6	2.36(1.31−4.27)	64.7	2.60(1.43−4.69)
Smoking status												
Never smoker	214		26.0	4.26(2.19−8.28)	41.2	3.24(1.96−5.36)	64.6	2.31(1.50−3.55)	65.2	2.67(1.73−4.13)	68.5	3.22(2.07−5.00)
Former smoker	210		19.4	2.70(1.36−5.37)	37.6	2.57(1.54−4.29)	69.9	2.95(1.88−4.63)	67.9	3.02(1.93−4.73)	67.9	3.16(2.01−4.96)
Current smoker	182		7.5	1	16.4	1	43.1	1	40.5	1	39.9	1
FTCD												
Low-Medium (0–5)	150		7.7	0.84(0.18−4.01)	19.7	–	46.2	2.33(0.93−5.82)	43.4	2.35(0.93−5.99)	44.1	3.30(1.22−8.93)
High (6–10)	32		6.9	1	0.0	–	27.6	1	26.7	1	20.7	1
Ever use of e-cigarettes												
No	546		19.5	3.53(1.14−10.97)	36.1	16.69(3.51−79.39)	61.7	1.81(1.03−3.16)	60.3	1.88(1.07−3.30)	61.7	2.03(1.16−3.57)
Yes	60		5.1	1	3.3	1	43.3	1	41.7	1	41.7	1

All ORs were adjusted for sex, age, and educational level.

FTCD: Fagerström test for cigarette dependence. OR: Odd Ratio; CI: confidence intervals.

Table 4 shows the proportion of people who were not in favor of the sale and marketing of e-cigarettes containing nicotine. 47.7% did not support the sale and marketing of e-cigarettes with nicotine, a percentage significantly higher among women (52.1%, OR = 1.50; 95%CI: 1.07–2.10), middle aged people (45–64 years old; 55.4%, OR = 1.90, 95%CI: 1.30–2.78) and older people (≧65 years old; 59.4%, OR = 2.16, 95%CI: 1.30–3.59). Smokers, particularly those with high scores in FTCD, and ever e-cigarette users were less likely to disagree with the sale and marketing of e-cigarettes with nicotine (table 4).

**Table 4:** Percentages (%), odds ratio (OR), and 95% confidence interval (95%CI) of people who are “disagree” and “totally disagree” with the commercialization of electronic cigarettes containing nicotine.

		n		%	OR (95%CI)
Overall		606		47.7	–
Sex					
Men		288		42.5	1
Women		318		52.1	1.50 (1.07−2.10)
Age					
≤44 years old		280		37.5	1
45–64 years old		216		55.4	1.90 (1.30−2.78)
≥65 years old		109		59.4	2.16 (1.30−3.59)
Educational level					
Low		71		59.7	1.47 (0.81−2.65)
Intermediate		236		52.0	1.36 (0.94−1.96)
High		299		41.4	1
Smoking status					
Never smoker		214		56.9	3.57 (2.27−5.62)
Former smoker		210		55.6	3.17 (1.99−5.04)
Current smoker		182		28.0	1
FTCD					
Low-Medium (0–5)		150		30.3	2.33 (0.85−6.40)
High (6–10)		32		18.2	1
Ever use of e-cigarettes					
No		546		52.0	12.56 (4.67−33.76)
Yes		60		8.6	1

All ORs were adjusted for sex, age, and educational level.

FTCD: Fagerström test for cigarette dependence. OR: Odd Ratio; CI: confidence intervals.

## Discussion

This is the first study to assess attitudes towards e-cigarette use in enclosed workplaces, public places (including public transports), and private venues (home and cars) in the general population. According to our data, approximately half of the general population did not agree with the use of e-cigarettes in any public place and indoor workplaces. The higher percentages of disagreement with their use were found for hospitals and other health care centers, and in schools (more than two thirds of the general population). However, lower percentages of disagreement with the use of e-cigarettes were found in the case of private venues (18.0% at homes and 32.5% in private vehicles).

We found that the disagreement with allowing the use of e-cigarettes in schools and in hospitals and health care centers (72% and 66% respectively) was similar to that observed towards extending smoking restrictions of conventional cigarettes to outdoors areas [19], [20]. We found a heterogeneous level of support to the use of e-cigarettes in all indoor areas studied between smokers and non-smokers; current smokers indicated less disagreement with the use of e-cigarettes. The differences between smokers and non-smokers were also found in other studies for the support of smoke-free legislation^21^ and the extension of smoking restrictions to outdoor areas [19], [22].

On the other hand, we surprisingly found similar degree of agreement with the use of e-cigarettes in all the public and private venues studied between current smokers and e-cigarettes users. This may be because the primary motivation of these potential users of e-cigarettes may be primarily to quit tobacco consumption or reduce the number of cigarettes smoked and not using these devices in public venues where smoking is banned. In this sense, one study conducted using an Internet panel of ever e-cigarette users showed that the main reasons for their use were the perception that they are less toxic than tobacco (84%) and the desire to quit smoking or avoid relapsing (77%), while only 34% declared to use the e-cigarette to avoid having to go outside to smoke [23]. Another study conducted by using an Internet panel of 19,000 e-cigarette users also showed that avoiding smoking bans in public places was the reason with the lowest score for initiating the e-cigarette use [24]. E-cigarettes, however, have been extensively marketed as a way to circumvent clean air policies in public places and enclosed workplaces [14]; this type of advertising could potentially increase dual use, because it may tend to be attractive to current cigarette smokers. Still, there is scarce evidence, quantitative and qualitative, about the real motivation for using e-cigarettes among their users in representative population samples.

Social acceptability of banning smoking in indoor public places, and consequently the support towards smoking regulations, has been an important issue for the politicians and legislators during the process of the implementation of smoke-free legislation worldwide, particularly in the hospitality sector [3], [25]. Similarly, our data showed a good social climate for promoting the restriction on using e-cigarettes in all workplaces and public places, including hospitality venues. According to the experiences of implementing indoor smoking restrictions in workplaces, the support for smoking bans increased after their implementation among the general population [26], smoker’ population [27], and for hospitality workers [28]–[30]. It is important to note that support for smoke-free legislation rises according to the level of tobacco control measures implemented in a particular country [31].

The tobacco industry has recently invested significantly in e-cigarettes, presumably because the sales of this new product has grown rapidly recent years [32] or as a strategy to undermine the public health gains of the last decades in tobacco control. The tobacco industry has always opposed smoke-free legislation and interfered during the debate around implementation of national smoke-free policies in various countries [33], [34]. The principal argument of the tobacco industry against the restriction of smoking in public places are that it threatens “individual freedom”, has negative economic impact in the hospitality sector, and displaces tobacco consumption from public to private venues, particularly the home. Scientific evidence has however rebutted all these hypotheses raised by the tobacco industry [35], [36].

Currently, there is scarce evidence about the mid- and long-term potential harmful health effects of e-cigarettes among users, particularly among non-users who are passively exposed [37]. However, the public health precautionary principle has led some governmental agencies, such as the US Food and Drug Administration, and the European Commission, to propose or adopt regulations for e-cigarettes. Furthermore, there is also a scientific debate about the ready availability of e-cigarettes to the population [38]–[40]. According to our data, 48% of the general population did not agree with the sale and marketing of e-liquids with nicotine. Certainly, there have been few, if any, quality controls on the manufacture of the e-cigarettes and their nicotine liquids to guarantee the safety or consistency of the product. Another important issue is the use of e-cigarettes in public places which could threaten the denormalization of tobacco use in indoor public places achieved through smoke-free legislation in the recent years [31], [41], [42]. For this reason, the WHO has recently called to the governments of the countries to restrict e-cigarettes use in all workplaces and public places, including the hospitality sector [43].

Some limitations to our study deserve consideration. The main limitations are the attrition of the cohort in the follow-up and the use of a questionnaire to collect the information. Regarding attrition, although there are not statistically significant differences between the people followed up and people lost according to sex, educational level, and smoking status, however the final sample overestimates the older people compared to the current distribution of the population in Barcelona We found systematically higher percentage of disagreement among the older population; for this reason, the prevalence of disagreement with the use of e-cigarette in indoor venues might be slightly overestimated. In fact, young people, particularly smokers, were those who showed less disagreement with the use of e-cigarettes in indoor venues. However, we tried to counteract this limitation by weighting the sample according to the age distribution of the population of Barcelona in 2013. On the other hand, we believe that our results underestimated the real attitudes toward allowing the use of e-cigarettes in all indoor venues because we found that 18% of our sample did not know what e-cigarettes are, and did not declare their attitudes towards e-cigarettes use. Moreover, there were statistically significant differences about knowledge on the e-cigarettes according to the age, level of education and current smoking status, being the oldest people (≧65 years old), people with low educational level, and never smokers, the strata of population with less knowledge about e-cigarettes (table 1). These strata of population indicated less agreement with allowing the use of e-cigarettes in indoor public places. Furthermore, the awareness of e-cigarettes is growing rapidly [6] and in a few years or months such knowledge will become universal. Hence, our results could be underestimating the likely attitudes toward allowing the use of e-cigarettes in indoor workplaces once knowledge about them is completely disseminated. Moreover, the different reactions about e-cigarettes regulation by the tobacco control community and public health authorities among different European countries could influence the public opinion in each country. For this reason, generalization of our results to other European countries should be cautious. The use of a questionnaire to collect self-reported information about the attitudes towards using e-cigarettes could be a source of bias. First, we used questions that measured the attitudes toward the use of e-cigarettes in indoors venues as proxy of the attitudes toward e-cigarette regulation. Second, we did not gather the reason why the people agreed or disagreed with the e-cigarette use in indoor places. More studies, particularly qualitative research, are needed to know in-depth the reasons why the general population agree or disagree with the e-cigarette use in public and workplaces. Nevertheless, strengths of our study include the fact that trained interviewers conducted a face-to-face interview at participants’ home, thus potentially increasing the internal validity of our results. In addition, this is the first time that information on the agreement of use of e-cigarettes is gathered among the general population in a European Country.

In conclusion, half of the general population did not support the use of e-cigarettes in workplaces and public places, including the hospitality sector. Moreover, the clear majority of the population (2 out of 3 people) disagreed with the use of e-cigarettes in hospital and other health care centers and in schools. Although there is a lack of scientific evidence about the harmful effects of passive exposure to the aerosols released or exhaled from e-cigarettes, avoiding the re-normalization of use of tobacco in indoor public places and a public health precautionary principle are strong arguments to promote e-cigarette regulation in all enclosed public venues without exception, as suggested by the WHO [43]. In this sense, our data show a favorable social climate that should be taken into account by legislators to extend e-cigarette regulation to all workplaces and enclosed public places in Spain.
